# Glioblastoma masquerading as a cystic brain lesion: A case report and evidence-based review

**DOI:** 10.1016/j.ijscr.2023.108277

**Published:** 2023-04-29

**Authors:** Moustafa A. Mansour, Dyana F. Khalil, Ahmad A. Ayad

**Affiliations:** a Department of Neurology and Neurologic Surgery, Faculty of Medicine, Al-Azhar University, Cairo, Egypt; b Department of Neurology and Neurologic Surgery, Mayo Clinic, Rochester, MN, USA; c Division of Neuro-Intensive Care, Dar Al-Fouad Medical Corporation, Cairo, Egypt; d Department of Emergency Medicine and Critical Care, Faculty of Medicine, Al-Azhar University, Cairo, Egypt; e Department of Oncology, Faculty of Medicine, Al-Azhar University, Cairo, Egypt

**Keywords:** Cystic brain lesion, Glioblastoma, Cystic glioblastoma, Neuroradiology, Case report

## Abstract

**Introduction and importance:**

In adults, glioblastomas account for approximately 12–15 % of primary intracranial neoplasms. In current standard-of-care treatment, glioblastomas have a 5-year survival rate of ~7.5 % and a median survival of ~15 months. Glioblastoma exhibits a highly variable imaging appearance, but the thick and irregular ring enhancement surrounding a necrotic core with infiltrative growth is the most prevalent imaging pattern. Glioblastoma with a cystic component (*also known as* cystic glioblastoma) is a rare presentation that can be misleading and often mistaken for other cystic brain lesions.

**Case presentation:**

In this report, we present a case of a 43-year-old woman who presented to the emergency department with a 2-month history of progressive neurologic manifestations that was attributed to a right-sided cystic brain lesion detected on routine imaging studies, which was later characterized as a cystic glioblastoma based on specific imaging and molecular studies.

**Clinical discussion:**

We highlight the importance of combining radiological and molecular modalities with clinical suspicion for a better characterization of cystic brain lesions and including glioblastoma in the list of potential diagnoses. Furthermore, we provide a comprehensive, evidence-based review of the entity of cystic glioblastoma and how the existence of the cystic component might affect the management and the overall prognosis.

**Conclusion:**

Several characteristics make cystic glioblastoma unique. However, it is also capable of mimicking other benign cystic brain lesions, delaying definitive diagnosis and hence the most appropriate management plan.

## Highlights

• Symptomatic cystic brain lesions are of an extended spectrum of potential etiologies.
• Infiltrating growth beyond the enhancing margin is a characteristic of glioblastomas.
• Glioblastoma has a molecular diagnosis based on wild-type IDH, ATRX, and Histone-3.
• Glioblastoma with cysts is a rare subtype of glioblastoma.
• A better prognosis for cystic over non-cystic glioblastomas is still controversial.

## 1 Introduction

Glioblastoma, previously known as glioblastoma multiforme (GBM), is the most aggressive primary brain tumor, accounting for approximately 15 % of all intracranial neoplasms and 49 % of malignant brain tumors [1]. Given whether a less malignant precursor lesion has or has not been involved in the tumor's progression course, glioblastomas can be classified into secondary or primary subtypes, with the primary tumors accounting for 95 % of all glioblastomas. The average incidence rate of glioblastoma is 3.19–4.17 per 100,000 population, with a male-to-female incidence ratio of almost one [2], [3], [4]. The median age for glioblastoma diagnosis is 64 years, but it can occur at any age [5]. Glioblastomas remain incurable despite decades of research and scientific advances, with a median survival of only 15–16 months with current standard treatments that consist of maximum safe surgical resection and chemoradiation [6], [7], [8]. The clinical symptoms of glioblastomas vary according to location, but seizures, focal neurologic deficits, and mental status changes are the most commonly reported symptoms [9]. Imaging studies remain the mainstay in diagnosing glioblastomas. However, glioblastomas have an extremely variable imaging appearance [10]. Glioblastomas typically exhibit a thick, irregular ring enhancement surrounding a necrotic core with an infiltrative growth pattern. A minor percentage of glioblastomas have been reported to present with one or more central cystic component(s) instead of, or in association with, a necrotic center. However, these cysts are more common in low-grade gliomas (e.g., juvenile pilocytic astrocytoma, ganglioglioma) and non-glial tumors such as hemangioblastoma. These glioblastomas with cystic component(s), *also known as* cystic glioblastomas, are a rare entity constituting only 7–23 % of all glioblastomas [11], [12]. Cystic glioblastomas present with radiological features mimicking other cystic brain lesions such as brain abscess, solitary metastasis, tumefactive demyelination, pilocytic astrocytoma, pleomorphic xanthoastrocytoma, or neuroglial cyst. Therefore, reaching the exact diagnosis can be very challenging, and more advanced diagnostic modalities may be required to make the definitive diagnosis, and hence the management plan. Herein, we report a case of a 43-year-old woman who presented to the emergency department with a 2-month history of headache, facial paresis, and left-sided paresthesia, with imaging studies revealing a cystic brain lesion. By using advanced imaging modalities, we could exclude several potential diagnoses to reach a preliminary diagnosis of cystic glioblastoma, which was confirmed later by the surgical biopsy results and tumor markers studies.

## 2 Case presentation

**Fig. 1:**
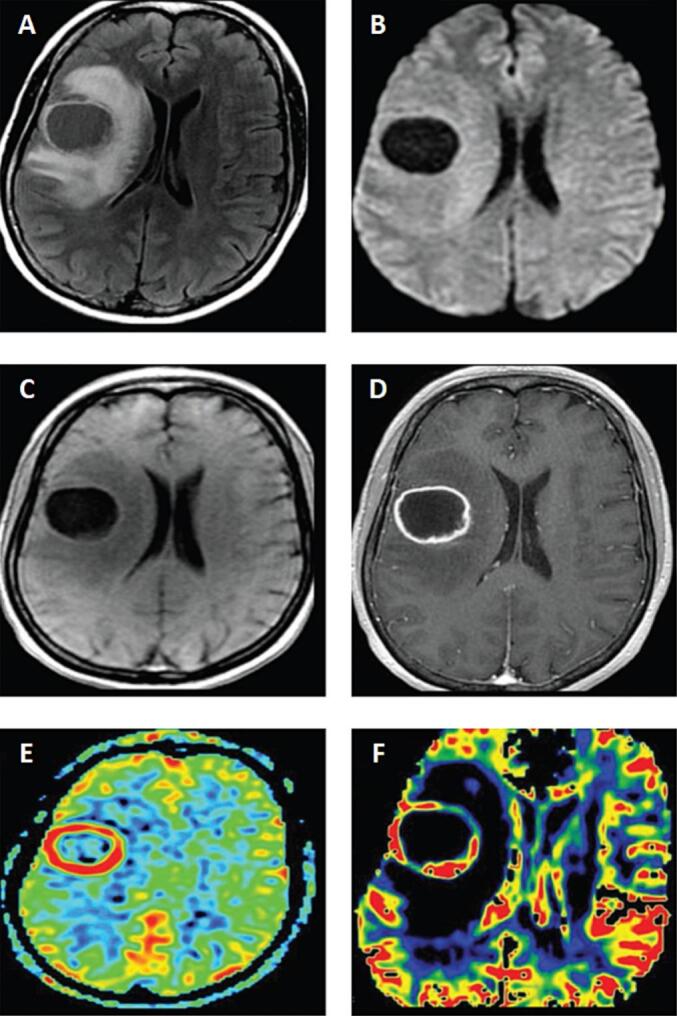
[A] Axial FLAIR and [B] axial DWI demonstrate a hypointense lesion with significant perilesional vasogenic edema and no restricted diffusion. [C] Axial precontrast T1WI and [D] axial postcontrast T1WI demonstrate a large irregular and thick ring-enhancing lesion in the right frontal lobe. [E] Perfusion without contrast, ASL, shows increased rCBF in the periphery of the lesion. [F] DSC perfusion MRI shows significantly elevated CBV at the periphery of the lesion.

**Fig. 2:**
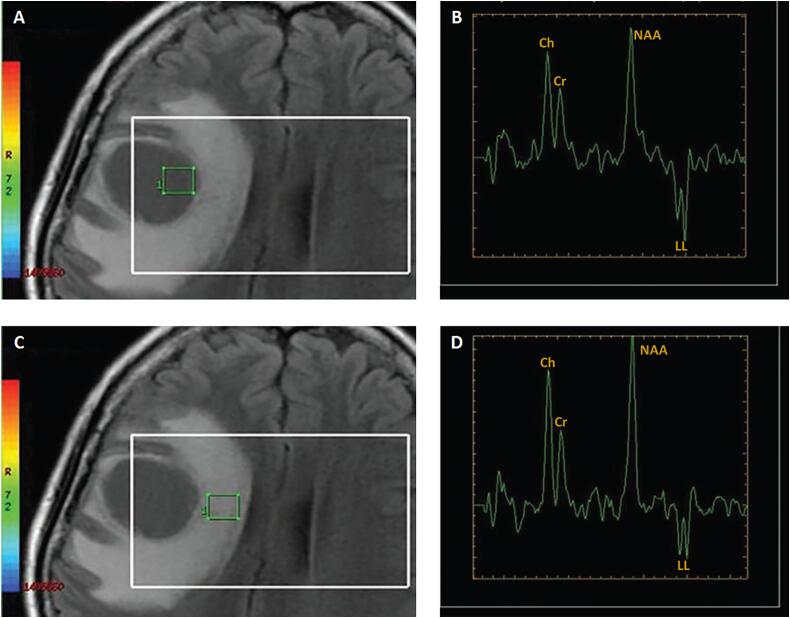
[A] Axial FLAIR localizer and [B] 3D multivoxel spectroscopy images demonstrate increased choline-to-creatine ratio, decreased NAA peak over the marginal component of the tumor, with a large inverted lactate/lipid peak. [C] Axial FLAIR localizer and [D] 3D multivoxel spectroscopy images demonstrate increased choline-to-creatine ratio and decreased NAA peak, with an inverted lactate/lipid peak also noted in the peritumoral area of the FLAIR abnormality beyond the enhancing component. (Ch: choline, Cr: creatine, NAA: *N*-acetylaspartate, LL: lactate/lipid.)

A previously healthy, immunocompetent 43-year-old woman presented to the emergency department (ED) with a 2-month history of progressive headache, behavioral changes, facial paresis, and left-sided paresthesia. She denied any history of fever, and no signs of meningism were evident. Hematological studies demonstrated normal findings. Radiological investigations revealed a hypointense lesion with significant perilesional vasogenic edema in the right frontal lobe, with partial effacement of the lateral ventricle on the same side (Fig. 1A). The lesion did not exhibit any diffusion restriction on the diffusion-weighted imaging (DWI) studies (Fig. 1B). Axial precontrast T1-weighted imaging (Fig. 1C) and axial postcontrast T1-weighted imaging (Fig. 1D) demonstrated a large irregular and thick ring-enhancing lesion in the right frontal lobe. Arterial spin labeling (ASL) MR perfusion studies showed increased relative cerebral blood flow (rCBF) in the periphery of the lesion (Fig. 1E). Dynamic susceptibility contrast (DSC) MR perfusion studies demonstrated significantly elevated cerebral blood volumes (CBV) in the boundaries of the lesion (Fig. 1F). Further investigations using magnetic resonance spectroscopy (MRS) (Fig. 2) demonstrated an increase in the choline-to-creatine ratio with a decrease in the NAA peak over the marginal component of the lesion, with an inverted lactate/lipid peak (Fig. 2A, B). Additionally, an increased ratio of choline-to-creatine with decreased NAA peak and inverted lactate/lipid peak were also noted in the peritumoral area of the FLAIR abnormality beyond the lesion-associated enhancing component (Fig. 2C, D).

**Fig. 3:**
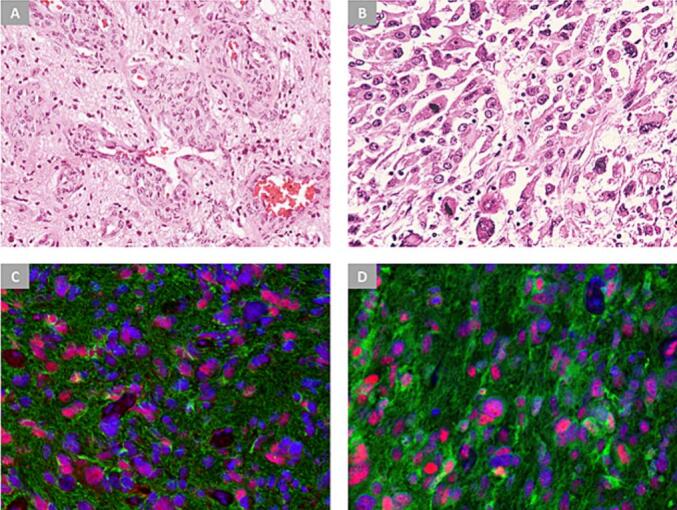
[A] Microscopic (H&E) image of the excised lesion shows microvascular proliferation with a glomeruloid-shaped configuration. [B] A higher magnification, microscopic (H&E) image demonstrates cellular anaplasia and dense mitotic figures. [C] Multiplexed IHC-IF staining study shows positive staining for wild-type ATRX (red) and GFAP (green) in tumor cells, DAPI is expressed in (blue). [D] Multiplexed IHC-IF staining study shows wild-type ATRX (red) and IDH1 (green) immunoreactivity in tumor cells.

Based on these findings, a preliminary diagnosis of glioma was made. Given that the patient was relatively young with a Karnofsky performance status (KPS) score of 60 (requires occasional assistance but is capable of caring for most of her needs), the patient underwent a right awake pterional (frontosphenotemporal) craniotomy, via which a good exposure of the lesion was achieved, and approximately 85–95 % of the lesion was safely resected. The surgery was uneventful and a biopsy specimen of the lesion was sent for histopathological examination (Fig. 3), which reported that the lesion was grade IV astrocytoma (glioblastoma) positive for wild-type isocitrate dehydrogenase-1 (IDH1) and alpha-thalassemia/mental retardation syndrome X-linked (ATRX) genes, with an associated atypical cystic component, consistent with the diagnosis of cystic glioblastoma. The patient was discharged two days later in a good general condition with a KPS score of 90, and subsequently referred to a radiation oncology specialist to start her regimen of chemoradiation. She tolerated a complete course of radiotherapy with concomitant temozolomide, followed by adjuvant temozolomide for five days a week every month. She recovered well and began to exhibit an improvement in her headaches, sensory functions, and facial expressions, and she was instructed to maintain regular follow-ups at the outpatient clinic for regular monitoring.

## 3 Case discussion

For a lesion to cause facial paresis, paresthesia, and personality changes, it is most likely to be located in the frontal lobe. Therefore, a right frontal lobe lesion was presumed in this patient based on her history and presenting manifestations. The progressive signs and symptoms indicate a chronic rather than an acute onset of the lesion. The first diagnostic test in such cases is a brain computed tomography (CT) scan because it is readily available in most healthcare facilities and can easily exclude acute life-threatening pathologies such as hemorrhages. Brain CT studies can be inconclusive on many occasions; therefore, a more advanced imaging modality, such as MRI, is often required. Symptomatic solitary cystic brain lesions can include an extended spectrum of potential diagnoses, as shown in Table 1.

**Table 1:** The most common etiologies of cystic brain lesions.

Type	Examples
Tumors	Solitary metastasis Juvenile pilocytic astrocytoma Pleomorphic xanthoastrocytoma Pilocytic astrocytoma Hemangioblastoma Central neurocytoma Ependymoma Medulloblastoma Ganglioglioma Dysembryoplastic embryonal tumor Cystic glioblastoma Lymphoma Craniopharyngioma Meningioma Pituitary macroadenoma Pineocytoma Schwannoma
Infections	Brain abscess Neurocysticercosis Tuberculoma Toxoplasmosis Chagas disease
Others	Tumefactive demyelination Neuroglial cyst Sarcoidosis Acute disseminated encephalomyelitis Vasculitis and Behcet disease

Given the patient's age, history, physical examination, laboratory and radiological investigations, and the location of the lesion, some of these listed etiologies could be easily excluded. For example, the patient's age and the frontal location of the lesion make juvenile pilocytic astrocytomas and medulloblastomas unlikely here, as they tend to affect younger patients with a predilection for the posterior fossa. The same applies to central neurocytomas and ependymomas that typically present as intraventricular lesions with/without increased intracranial tension, and gangliogliomas that have a temporal lobe predilection (~70 %) and are typically associated with enhancing mural nodules when present as cystic lesions. Therefore, we were able to narrow down the differential diagnosis spectrum for our patient to what is shown in Table 2 [13], [14], [15].

**Table 2:** The most likely etiologies of the patient's cystic brain lesion.

Type	Examples
Tumors	Solitary metastasis Pleomorphic xanthoastrocytoma Pilocytic astrocytoma Hemangioblastoma Cystic glioblastoma Lymphoma
Infections	Brain abscess Tuberculoma
Others	Tumefactive demyelination Neuroglial cyst

Given the absence of systemic and neurologic signs of infection on top of diffusion restriction at the lesion core in this patient, we could rule out pyogenic abscesses and tuberculomas from this list [16]. Furthermore, the increased perfusion parameters at the lesion margin were typical for an angiogenic tumor rather than a pyogenic lesion or a hypercellular tumor (e.g., lymphoma); however, some abscesses have been reported to be associated with subtly elevated perfusion parameters, such as some fungal abscesses [16], [17]. Brain metastases are typically multiple and peripherally located, but solitary brain metastases are not uncommon. However, the nearly 100 % signal recovery and tumoral spectral pattern beyond the enhancing component of the tumor, especially choline levels, were suggestive of an infiltrative tumor rather than a metastasis or a pure vascular tumor (e.g., hemangioblastoma) in this patient [18], [19]. Tumefactive demyelination could be easily excluded in this patient by the presence of complete rather than incomplete ring enhancement, significantly elevated perfusion parameters, and the infiltrative nature of the lesion [20], [21]. Also, the increased perfusion parameters and the infiltrative growth pattern were not features of pilocytic astrocytomas (WHO grade 1) or pleomorphic xanthoastrocytomas (WHO grade 2) [22], [23]. Neuroglial cysts typically lack enhancement and increased perfusion on imaging [24], [25], but the opposites of these features were evident in this patient. Additionally, the significantly increased CBF (evidenced by ASL images) and CBV (evidenced by DSC perfusion images) were suggestive of increased neoangiogenesis with leakiness of the blood vessels that are typically seen in gliomas (mostly glioblastomas) [22], [26]. Furthermore, the presence of a large area of central necrosis with the suggestive spectroscopy findings confirms the diagnosis of glioma in our case.

**Fig. 4:**
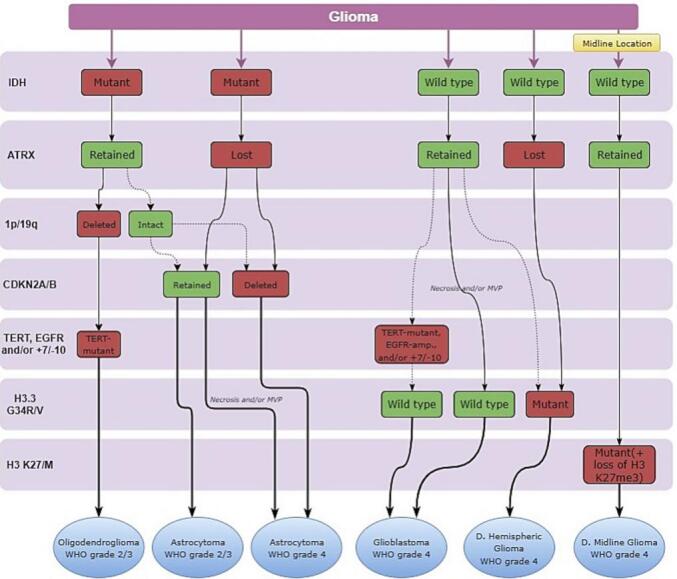
A schematic illustration of the seven-layer approach to the classification of diffuse gliomas in adults. The presence and absence of the diagnostically most relevant molecular alterations for each tumor type are highlighted in green and red, respectively. (MVP: microvascular proliferation, D: diffuse.)

Gliomas are known to be heterogeneous because they change and adapt in response to the composition of their microenvironment and the therapy used. Therefore, developing molecular classifiers that provide meaningful ways to stratify patients remains a major challenge for the field. Since 2008, the classification of brain tumors by the World Health Organization (WHO) has evolved to date with the latest fifth edition (WHO CNS5, 2021) [27]. The updated classification emphasizes the need for a classification of gliomas based on histological and molecular genetic features for clinical diagnosis and outcome prediction. With the WHO CNS5 2021 edition, several IHC diagnostic and prognostic markers have been added to the diagnostic criteria of gliomas using a 7-layer algorithm that combines histological features, grading, and molecular information [28]. An illustration of the new seven-layer approach for glioma classification is demonstrated in (Fig. 4). By applying this method to the patient's tumor specimen, we were able to observe dual immunoreactivity of wild-type ATRX and IDH1 in tumor cells, using multiplexed IHC-IF staining technique (Fig. 3C, D), in addition to a wild-type Histone 3 as confirmed by negative results for G34R/V and K27/M mutations (not shown), further confirming the diagnosis of glioblastoma in this patient.

Glioblastoma is the most frequent and malignant histological type, accounting for the majority of gliomas with an exceedingly poor 5-year survival rate. Glioblastomas are relatively resistant to standard-of-care therapy compared to other brain neoplasms. They typically appear as heterogeneous white matter lesions with irregular peripheral enhancement post-contrast, besides a necrotic core and surrounding vasogenic edema [29]. Treatment primarily includes surgery with concurrent chemoradiation, but they inevitably recur. Rather than describing a specific pathological category, cystic glioblastoma refers to a subtype of glioblastoma with one or more cystic components. Cystic glioblastomas are relatively rare compared to other forms of glioblastomas. Several studies have reported a survival benefit in patients with cystic glioblastomas and fewer relapses than those without cysts, but other studies have reported no survival benefit from cystic glioblastoma [30], [31], [32], [33], [34], [35], [36]. Due to the relatively small sample size in these studies, this controversy and the lack of statistical significance might be explained by the lack of statistical power. Thus, a well-designed prospective study with more patients would provide more conclusive evidence on the survival benefit of the cystic component in glioblastoma patients.

## 4 Conclusions

• Glioblastoma is the most frequent and malignant brain tumor, with an exceedingly poor survival rate.
• Cystic glioblastoma represents a rare subtype of glioblastomas characterized by one or more cystic components.
• Several characteristics make cystic glioblastomas unique. However, they are also capable of mimicking other lesions, which can lead them to be mistaken for other benign cystic brain lesions, hence delaying the most appropriate management.
• It is imperative that a symptomatic solitary cystic brain lesion is evaluated with advanced imaging techniques to better determine the underlying etiology and the most appropriate treatment plan.
• Cystic glioblastomas may have a better prognosis than non-cystic glioblastomas, but evidence needs to be obtained from well-designed, statistically significant studies.

## Methods

The work presented in this manuscript has been reported in line with the SCARE criteria [37].

## Ethical approval

Ethical approval for this study (Ref# HZU_22_02136) was provided by the Ethical Committee HZU of Al-Azhar University Hospitals, Cairo, Egypt on 15 January 2022. The patient provided a written informed consent to publish the case and any related data.

## Funding

None declared.

## Guarantor

Dr. Moustafa A. Mansour (the corresponding author).

## CRediT authorship contribution statement

**M.M.** was responsible for the conception of the work, data collection, drafting the article, critical revisions, illustrating the figures, and obtaining approval of the final version of the manuscript. **D.K.** contributed by drafting the article, and critical revisions. **A.A.** contributed by critical revisions of the article. All authors read the final manuscript and were involved in direct patient care.

## Competing interest

None declared.
